# Erythema Nodosum in a Child with Celiac Disease

**DOI:** 10.1155/2011/935153

**Published:** 2011-09-13

**Authors:** Andrew Fretzayas, Maria Moustaki, Olga Liapi, Polyxeni Nicolaidou

**Affiliations:** 3rd Department of Pediatrics, School of Medicine, “Attikon” University Hospital, University of Athens, 12462 Athens, Greece

## Abstract

Erythema nodosum is an acute, nodular, erythematous eruption usually limited to the extensor aspects of the lower legs. It could be idiopathic or associated with other systemic diseases. We, herein, report a phenotypically healthy, ten-year-old boy who presented with erythema nodosum in whom serological tests of autoimmunity and intestinal histological examination were compatible with celiac disease. The eruption resolved within 2 months following a gluten-free diet. Therefore, the possibility that erythema nodosum represents an extraintestinal manifestation of celiac disease should be kept in mind accordingly in cases where other common causes of this rash are ruled out.

## 1. Introduction

Celiac disease is an immune-mediated enteropathy provoked by permanent sensitivity to gluten in genetically susceptible individuals [1]. The typical presentation of the disease is characterized by gastrointestinal symptoms. Extraintestinal features are the main manifestations of the atypical form. Furthermore, silent and latent or potential forms of the disease have been recognized [1]. 

Mucocutaneous manifestations are quite common among children diagnosed with celiac disease [2]. However, erythema nodosum has rarely been described [3, 4]. We, herein, report a child who presented with this intriguing appearance and was diagnosed as having celiac disease.

## 2. Patient

A 10-year-old boy was referred to our department having tender nodular, erythematous eruption limited to the extensor aspects of the lower legs. The eruption manifested four weeks prior to the admission. There were no other symptoms such us fever, coryza, malaise, or gastrointestinal symptoms, and he was not on any medication. The physical examination was unremarkable with weight and height being on the 25th centile. However, from his growth records, it was shown that at the age of 3 years his weight and height were on the 75th centile and since then they declined gradually to the 25th centile. He was diagnosed on clinical grounds as having erythema nodosum, and an extensive workup was undertaken. 

The initial investigation included a full blood count which was normal for his age (hemoglobin 12.5 g/dL, hematocrit 38%, white blood cells 4980/*μ*L with normal differential, platelets 339.000/*μ*L); erythrocyte sedimentation rate and C-reactive protein were 4 mm/h and 1 mg/L, respectively. A throat swab culture revealed normal flora, whereas antistreptolysin O and anti-DNase-B were within the normal range. Stool cultures did not isolate any pathogen. Mantoux was negative and a chest X-ray was normal with no indication for hilar or mediastinal lymphadenopathy. Abdominal ultrasound did not reveal any pathology. The biochemistry studies showed alkaline phosphatase (269 U/L) with normal liver enzyme activities and marginally low phosphorus (3.6 mg/dL). Complement profile, immunoglobulins, and serum angiotensin-converting enzyme activity were also within normal range. 25-OH-vitamin D was 29.3 ng/mL, parathyroid hormone was 32.8 pg/mL, and urine Calcium/Creatinine was 0.08. The estimation of bone mineral density (BMD) of the lumbar region, using the dual energy X-Ray absorptiometry (DEXA) method, revealed BMD ( L1–L4) 0.606 g/cm^2^ (z score = −0.57) 

As the most common causes of erythema nodosum were ruled out, the investigation was directed to infrequent causes such as celiac disease. IgA antibodies to tissue transglutaminase (anti t-TG) were elevated (43 IU), and HLA typing revealed that the child had DQ2 haplotype. Duodenoscopy was performed and histological examination of distal duodenal specimens was compatible with celiac disease. 

 Following a gluten-free diet, the eruption, which has already persisted for 2 months, resolved within the next two months. One year later, anti-t-TG antibodies became negative whereas a three-year follow up was uneventful with no recurrence of the rash. During this three-year period, his weight raised from the 25th to the 75th centile and his height from the 25th to the 50th centile.

## 3. Discussion

Herein, we presented a boy with erythema nodosum who was diagnosed as having celiac disease. The spectrum of skin manifestations in celiac disease is wide [2], and they belong to extraintestinal manifestations of the disease. 

Erythema nodosum associated with celiac disease was twice reported [3, 4] since 1991. The first reported case [3] was a 17-year-old woman with a 6-month history of recurrent erythema nodosum associated with episodes of diarrhea and megaloblastic anemia. The erythema subsided after the establishment of celiac disease diagnosis and the treatment of the patient with a strict gluten-free diet. The second reported case [4] was also a young woman (16 years old) with a history of recurrent erythema nodosum lasting 4 years. At the age of 2 years, she had been treated for acute lymphoblastic leukemia and at the age of 14 years for hemangioblastoma with no recurrence since then. Because of a low serum iron, she was investigated for malabsorption syndromes and she was found to have celiac disease. The erythema resolved within one month after the initiation of a gluten-free diet, and it recurred for a short period only, when the patient was unintentionally exposed to gluten. It was postulated that the increased intestinal permeability to antigens may provoke the skin hypersensitivity reaction. 

Furthermore, it should be noticed that celiac disease may coexist with sarcoidosis [5] which is a common cause of erythema nodosum. Actually, the coincidence of sarcoidosis, celiac disease, and erythema nodosum [6] has been described but at that time erythema was attributed to sarcoidosis. Erythema nodosum may therefore not be such a rare manifestation of celiac disease. It may be on the contrary that this diagnosis is overlooked in children with erythema nodosum given that celiac disease can be atypical or latent without pronounced signs of malabsorption [1]. Our patient belonged to this category as, with the exception of erythema nodosum, he had no other symptoms. It could be therefore classified to the silent form of celiac disease until the development of the eruption given that the intestine on the histological examination was damaged. 

It should be noted that although the child had weight and height within the normal range (25th centile), before being on gluten-free diet, his height centile increased to the 50th after following a gluten-free diet. Therefore, his height at presentation was below his potential. 

In general, erythema nodosum resolves within 5–8 weeks time. Most lesions in infection-induced erythema nodosum heal within 7 weeks, but active disease may last up to 18 weeks [7]. The disease may last for a long period when the antigenic stimulus persists. In our patient, the duration of the rash was not very long as the patient was put on a gluten-free diet without delay after the establishment of the underlying disease. 

In conclusion, celiac disease should be investigated in any child with erythema nodosum of unknown etiology even when there are no other symptoms or signs indicative of the disease or skin lesions are persistent.
